# *ALKBH5* modulates m6A modification to enhance acute myeloid leukemia resistance to adriamycin

**DOI:** 10.17305/bb.2024.11076

**Published:** 2024-10-27

**Authors:** Yonghua Liu, Jinhong Jiang, Yuxiao Zeng, Yu Jiang

**Affiliations:** 1 Department of Hematology, Sixth Affiliated Hospital of Wenzhou Medical University, Lishui, China

**Keywords:** *ALKBH5*, *TUG1*, *SH3BGRL*, *EHMT2*, acute myeloid leukemia, N6-methyladenosine, H3K9me2

## Abstract

Acute myeloid leukemia (AML) is a fatal malignancy with rising incidence and low cure rates. This study aims to investigate the effect of alkB homolog 5 (*ALKBH5*)-mediated N6-methyladenosine (m6A) modification on adriamycin (ADR) resistance in AML. First, the levels of *ALKBH5*, taurine upregulated 1 (*TUG1*), YTH N6-methyladenosine RNA binding protein F2 (*YTHDF2*), euchromatic histone lysine methyltransferase 2 (*EHMT2*), and SH3 domain-binding glutamate-rich protein-like (*SH3BGRL*) were measured. IC50 values, cell proliferation, and apoptosis were determined. m6A levels were analyzed, and the binding interactions between *TUG1* and YTHDF2, as well as *TUG1* and EHMT2, were assessed. The stability of *TUG1* and the enrichment of EHMT2 and H3K9me2 on the *SH3BGRL* promoter were confirmed. *In vivo* experiments were conducted to further validate the results. The findings revealed that *ALKBH5* was overexpressed in both AML- and ADR-resistant cells, and silencing *ALKBH5* reduced the ADR resistance of AML cells. *ALKBH5* removed m6A modifications from *TUG1*, disrupting the interaction between YTHDF2 and *TUG1*, thereby stabilizing *TUG1* expression. *TUG1* bound to EHMT2, promoting H3K9me2 modification on the SH3BGRL promoter and suppressing SH3BGRL expression. Overexpression of *TUG1* or knockdown of *SH3BGRL* reversed the suppressive effect of *ALKBH5* knockdown on ADR resistance. *In vivo*, *ALKBH5* knockdown inhibited ADR resistance in AML cells. In conclusion, ALKBH5 removed m6A modification to stabilize *TUG1* expression in a YTHDF2-dependent manner, enhancing H3K9me2 levels on the *SH3BGRL* promoter and suppressing *SH3BGRL* expression, thus promoting ADR resistance in AML cells.

## Introduction

Leukemia, arising from bone marrow and blood, is an aggressive and often deadly cancer characterized by the carcinogenic transformation of leukocytes, which ultimately leads to a severe decline in immune function [1]. Acute myeloid leukemia (AML), a predominant type of leukemia, represents malignancy within the myeloid lineage stem cells, resulting in high mortality and challenging treatment complications [2]. Recently, an increasing number of drugs have been adopted in AML treatment, improving the overall survival rate of affected patients [3]. For instance, adriamycin (ADR), a prominent anthracycline anti-cancer drug, is commonly used for tumor remission due to its significant impact on DNA repair, cancer cell activity, hematopoiesis, and immune function [4]. However, despite ADR’s clinical benefits, the rapid rise of ADR resistance has become a pressing and complex challenge in AML therapy [5]. Therefore, it is crucial to identify effective biomarkers that can reduce ADR resistance and enable tailored treatment options for AML patients.

N6-methyladenosine (m6A) modification is a dynamic process involved in various carcinogenic activities, including hematopoiesis, metabolism, mRNA translation, and immune function [6]. Recent studies have shown that m6A modification is mediated by YTH N6-methyladenosine RNA binding protein F (YTHDF) and reduced by alkB homolog 5 (*ALKBH5*) across multiple cancers [7]. *ALKBH5*, an important m6A demethylase, is abnormally expressed in AML, where it sustains leukemia stem cell activity and affects hematopoiesis [8]. Furthermore, *ALKBH5* is recognized as an oncogene in cancer progression, contributing to cellular resistance to chemotherapy [9]. Notably, *ALKBH5* interacts with a broad range of long non-coding RNAs (lncRNAs) to modulate malignant processes [10]. LncRNAs play significant roles in cancer progression and treatment as tumor suppressors or promoters, influencing gene expression, modulating protein transcription, and establishing molecular interactions [11]. One such lncRNA, taurine upregulated 1 (*TUG1*), has gained attention as a biomarker in cancer detection and prognosis due to its role in cell survival, mobility, transformation, aggressiveness, apoptosis, and drug sensitivity [12]. *TUG1* is abundantly expressed in AML tissues, and its depletion reduces drug resistance in AML therapy [13]. Previous studies suggest that *TUG1* may regulate downstream gene expression in cancer progression [14], prompting further exploration into *TUG1*’s potential targets. Of interest, the SH3 domain-binding glutamate-rich protein-like (*SH3BGRL*), a scaffold protein expressed in various human tissues, is downregulated in AML and enhances drug sensitivity in AML patients [15].

This study aims to elucidate the specific mechanism by which *ALKBH5* mediates m6A modification through the *TUG1/SH3BGRL* pathway, contributing to ADR resistance in AML cells, thereby providing a new theoretical foundation for improving AML treatment.

## Materials and methods

### Cell culture and treatment

Human AML cell lines [HL60 (SNL-040), SNL-040 (SNL-040), HEL (SNL-045), and K562 (SNL-042)] and the human embryonic kidney cell line HEK293T (SNL-015) (all obtained from Wuhan Sunncell Biotechnology, Wuhan, Hubei, China) were cultured in Roswell Park Memorial Institute 1640 medium (11875093, Gibco, Carlsbad, CA, USA) supplemented with 10% fetal bovine serum (FBS, 10099158) and 1% penicillin/streptomycin (15140148) (both from Gibco) at 37 ^∘^C with 5% CO_2_. AML-resistant cell lines (HL60/ADR and KG-1/ADR) were established as previously described [16]. Briefly, parental HL60 and KG-1 cells were cultured with ADR (D1515, Sigma, St. Louis, USA) at incrementally increasing concentrations (1–5 mg/L) over six months until ADR treatment had no significant effect on cellular morphology or proliferation. Cells were subsequently maintained in 0.5 µM ADR to preserve the drug-resistant phenotype of HL60/ADR and KG-1/ADR cells.

### Cell transfection

Small interfering RNAs (siRNAs) targeting *ALKBH5* (si-ALKBH5-1, si-ALKBH5-2, and si-ALKBH5-3), *YTHDF2* (si-YTHDF2-1, si-YTHDF2-2, and si-YTHDF2-3), and *SH3BGRL* (si-SH3BGRL-1, si-SH3BGRL-2, and si-SH3BGRL-3), as well as siRNA negative control (NC), overexpression plasmids for TUG1, euchromatic histone lysine methyltransferase 2 (*EHMT2*), and oe-NC were purchased from Sangon Biotechnology (Shanghai, China). Lentivirus-packaged short hairpin RNAs (shRNAs) targeting NC and *ALKBH5* were purchased from Hanbio Biotechnology (Shanghai, China). Cells were transfected using Lipofectamine 3000 (L3000015, Thermo Fisher, Waltham, MA, USA) according to the manufacturer’s instructions. After 48 h, transfection efficiency was assessed, and further experiments were conducted. siRNA and shRNA sequences are provided in Table 1.

**Table 1:** Sequence of siRNAs and shRNA

**siRNA**	**Target position**	**Sequence**
si-ALKBH5-1	1100	SS	GCUGCAAGUUCCAGUUCAAGC
		AS	UUGAACUGGAACUUGCAGCCG
si-ALKBH5-2	980	SS	GCGCCGUCAUCAACGACUACC
		AS	UAGUCGUUGAUGACGGCGCUG
si-ALKBH5-3	1780	SS	GGACCUAGGUUCUCAUAUUCU
		AS	AAUAUGAGAACCUAGGUCCUG
si-YTHDF2-1	845	SS	GCACAGAAGUUGCAAGCAAUG
		AS	UUGCUUGCAACUUCUGUGCUA
si-YTHDF2-2	2496	SS	GGAGAAUAUAACAGUGUUACC
		AS	UAACACUGUUAUAUUCUCCUA
si-YTHDF2-3	2629	SS	GGAUUAAUUUGAUUUCAAAGC
		AS	UUUGAAAUCAAAUUAAUCCUG
si-SH3BGRL-1	292	SS	GGAUCAAGAUGGUGAAAUAGA
		AS	UAUUUCACCAUCUUGAUCCAU
si-SH3BGRL-2	445	SS	GAUUAAGAAGAAACAACAAGA
		AS	UUGUUGUUUCUUCUUAAUCUG
si-SH3BGRL-3	836	SS	GCUUAAUGUUGAAAUAAUAGA
		AS	UAUUAUUUCAACAUUAAGCCU
sh-ALKBH5	1102	SS	GCTGCAAGTTCCAGTTCAA
		AS	TTGAACTGGAACTTGCAGC

si: Small interfere; sh: Short hairpin; *ALKBH5*: AlkB homolog 5; *YTHDF2*: YTH N6-methyladenosine RNA binding protein F2; *SH3BGRL*: SH3 domain binding glutamate rich protein like; siRNA: Small interfering RNA; shRNA: Short hairpin RNA.

### Cell counting kit-8 (CCK-8) assay

The IC50 value of ADR was determined using the CCK-8 assay. Transfected cells were seeded in 96-well plates, treated with ADR at various concentrations (0.1, 0.5, 1, 2, 4, 8, 16, and 32 µM) for 48 h, and incubated with 10 µL of CCK-8 reagent (CK04, Dojindo Laboratories Co., Ltd., Kumamoto, Japan) for 3 h. The optical density (OD) at 450 nm was measured with a microplate reader. The cell growth curve was plotted, with the IC50 value representing a 50% inhibition of cell viability.

### Colony formation assay

Cells were seeded into six-well plates (1 × 10^3^ cells/well) and incubated at 37 ^∘^C for two weeks. Colonies were fixed in methanol, stained with 0.1% crystal violet, imaged, and counted.

### Terminal deoxynucleotidyl transferase-mediated dUTP nick end labeling (TUNEL) assay

For apoptosis detection, cells (1 × 10^ImEquation2^) were resuspended in 0.5 mL phosphate-buffered saline (PBS) and processed according to the TUNEL kit instructions (ab66108, Abcam Inc., Cambridge, MA, USA). Cells were imaged under a fluorescence microscope (LSM700B, Zeiss, Oberkochen, Germany).

### Quantitative real-time polymerase chain reaction (qRT-PCR)

Total RNA was extracted from cells and tumor tissues using Trizol Reagent (Invitrogen, Carlsbad, CA, USA), and RNA quality and concentration were measured with a NanoDrop ND-1000 spectrophotometer (NanoDrop Technologies, Montchanin, DE, USA). Complementary DNA (cDNA) was synthesized using random primers (Fermentas, St. Leon-Rot, Germany) and M-MLV reverse transcriptase (Invitrogen) and quantified with SYBR Green Master Mix (Applied Biosystems), using glyceraldehyde-3-phosphate dehydrogenase (*GAPDH*) or U6 as internal reference genes [17]. Relative expression levels were calculated by the 2^-ΔΔCt^ method. Primer sequences are listed in Table 2.

**Table 2:** Primer sequence of qRT-PCR

	**Forward primer (5′--3′)**	**Reverse primer (5′--3′)**
*ALKBH5*	ACGGATCCTGGAGATGGACA	ATCTTCACCTTTCGGGCAGG
*TUG1*	TAGCAGTTCCCCAATCCTTG	CACAAATTCCCATCATTC
*YTHDF2*	ATAGTTTGCCTCCAGCCACC	CTTTTGCAACGGGACCCTTG
*EHMT2*	ACCATGACTGCGTGCTGTTA	CGGTTGAGTTGAAGCGCAAA
*SH3BGRL* promoter	TTCTCCTCGCCCTCTTCTCA	CTGCAGTTCCAGCCCAAAAC
*SH3BGRL*	CCCCTGCCACCTCAGATTTT	GCTTTGCTTGCACTTCTGCT
*GAPDH*	GATTCCACCCATGGCAAATTC	CTGGAAGATGGTGATGGGATT
*U6*	AAAATATGGAACGCTTCACGAA	AAAATATGGAACGCTTCACGAA

qRT-PCR: Quantitative real-time polymerase chain reaction; *ALKBH5*: AlkB homolog 5; *TUG1*: Taurine up-regulated 1; *YTHDF2*: YTH N6-methyladenosine RNA binding protein F2; *EHMT2*: Euchromatic histone lysine methyltransferase 2; *SH3BGRL*: SH3 domain binding glutamate rich protein like; *GAPDH*: Glyceraldehyde-3-phosphate dehydrogenase.

### Western blot analysis

Proteins were extracted from cells and tumor tissues with the protein extraction kit (BC3710, Solarbio, Beijing, China). Protein samples were separated on 10% SDS-PAGE gels, transferred onto polyvinylidene fluoride membranes, blocked with 5% skim milk, and incubated with primary antibodies (rabbit anti-ALKBH5, 1:1000, ab195377; rabbit anti-YTHDF2, 1:1000, ab220163; rabbit anti-EHMT2, 1:1000, ab229455; and rabbit anti-SH3BGRL, 1:500, 11253-1-AP) overnight at 4 ^∘^C. Membranes were washed and incubated with goat anti-rabbit IgG secondary antibody (1:5000, ab205718). Target protein expression was visualized using enhanced chemiluminescence substrate (PE0010, Thermo Fisher), with GAPDH as the loading control.

### Total m6A level determination

Total RNA was extracted, and m6A levels were measured using the m6A RNA Methylation Quantification Kit (ab185912, Abcam). Briefly, 200 ng RNA and 80 µL Binding Solution were added to 96-well plates and incubated at 37 ^∘^C for 90 min. Wells were then treated with Capture Antibody for 60 min at room temperature, Detection Antibody, and Enhancer Solution, followed by Developer Solution in the dark. Absorbance was measured at 450 nm. The percentage of m6A in total RNA was calculated as follows: m6A% ═ [(Sample OD - NC OD) ÷ S]/[(PC OD - NC OD)] × 100%, where NC and PC are the negative and positive controls, *S* is the sample RNA amount, and *P* is the positive control RNA amount.

**Figure 1.**
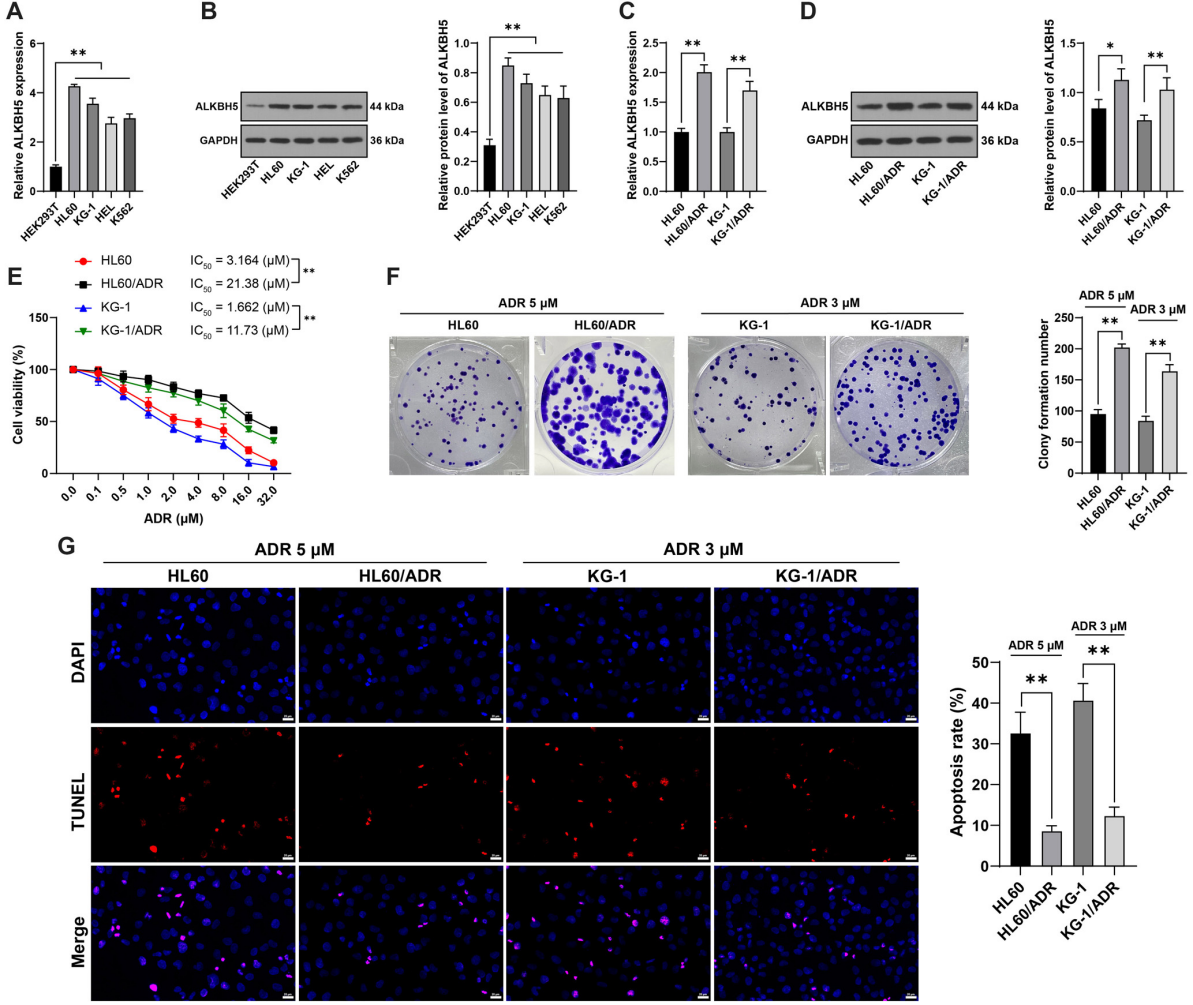
***ALKBH5* is overexpressed in AML ADR-resistant cells.** Panels (A and B): The expression of *ALKBH5* in the human embryonic kidney cell line HEK293T and AML cell lines (HL60, KG-1, HEL, and THP-1) was evaluated using qRT-PCR (A) and western blot analysis (B). Panels (C and D): *ALKBH5* expression in the established ADR-resistant AML cell lines HL60/ADR and KG-1/ADR was determined by qRT-PCR (C) and western blot analysis (D). Panel (E): Cell viability and the IC50 value for ADR were assessed using the CCK-8 assay. Panel (F): Cell proliferation was evaluated through a colony formation assay. Panel (G): Apoptosis was measured using TUNEL staining. All experiments were conducted independently in triplicate, and data are presented as mean ± standard deviation. One-way ANOVA was applied to analyze the data in panels (A–D, F, and G), while two-way ANOVA was used for panel (E). Tukey’s multiple comparisons test was utilized for post hoc analysis. *P < 0.05, **P < 0.01. *ALKBH5*: AlkB homolog 5; ADR: Adriamycin; CCK-8: Cell counting kit-8; TUNEL: Transferase-mediated dUTP nick end labeling; AML: Acute myeloid leukemia; qRT-PCR: Quantitative real-time polymerase chain reaction.

### RNA immunoprecipitation (RIP) assay

Total RNA was extracted, and RIP was performed using m6A antibody (1:50, ab208577), YTHDF2 antibody (1:30, ab220163), or IgG antibody (1:50, ab172730) (all from Abcam) bound to protein A/G magnetic beads in IP buffer (140 mM NaCl, 1% NP-40, 2 mM EDTA, 20 mM Tris, pH 7.5) at 4 ^∘^C overnight. Immunoprecipitated RNA was eluted and analyzed by qRT-PCR to assess *TUG1* levels. Primer sequences are listed in Table 2.

**Figure 2.**
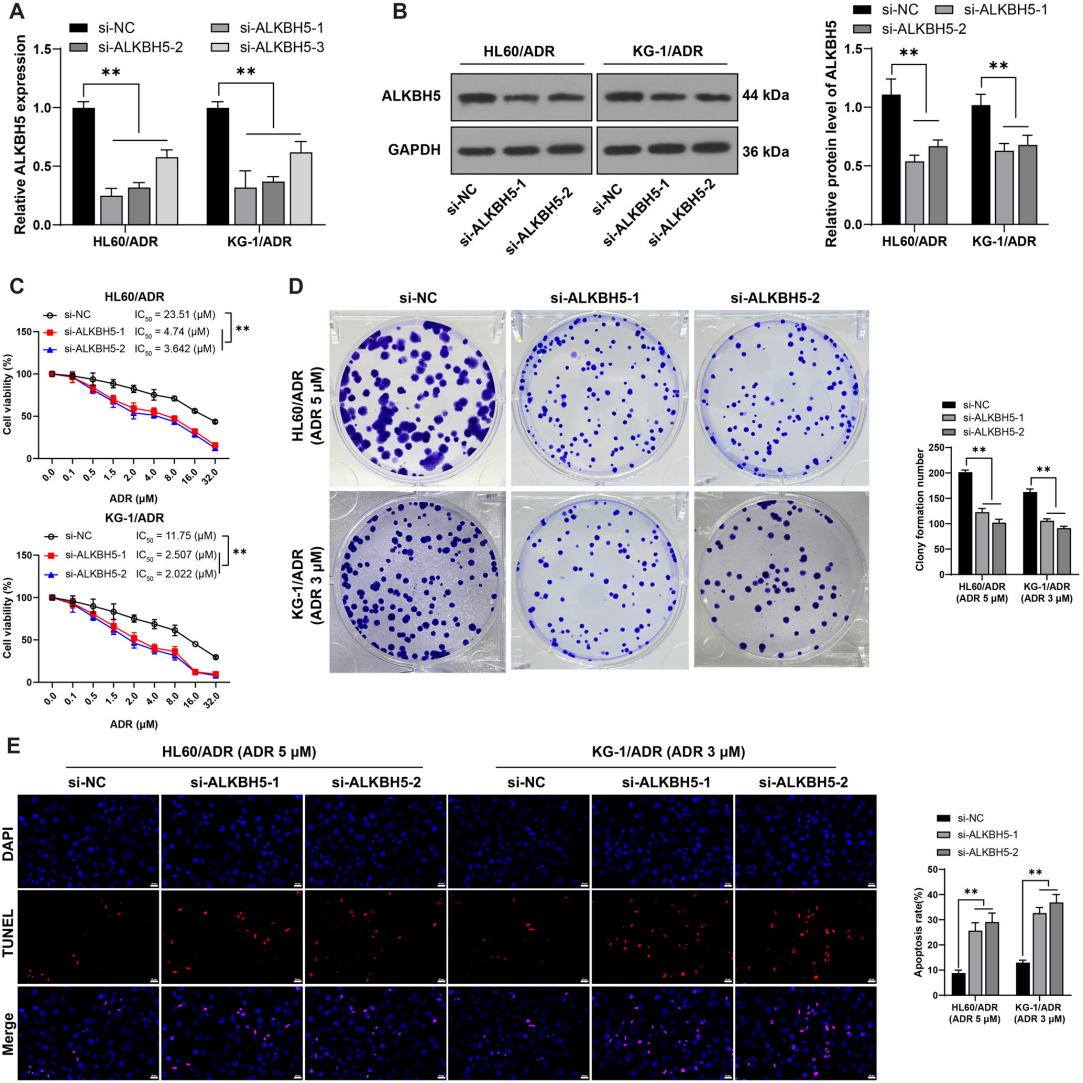
**Inhibition of *ALKBH5* downregulates ADR resistance in HL60/ADR and KG-1/ADR cells**. si-ALKBH5 constructs (si-ALKBH5-1, si-ALKBH5-2, and si-ALKBH5-3) were transfected into HL60/ADR and KG-1/ADR cells, with si-NC serving as the control. Panel (A): Transfection efficiency was evaluated by qRT-PCR, with si-ALKBH5-1 and si-ALKBH5-2 chosen for subsequent experiments due to their higher transfection efficiency. Panel (B): *ALKBH5* expression levels were assessed by western blot analysis. Panel (C): Cell viability and the IC50 for ADR were measured using the CCK-8 assay. Panel (D): Cell proliferation was examined through colony formation assays. Panel (E): Apoptosis was quantified using TUNEL staining. All experiments were independently repeated three times, and data are presented as mean ± standard deviation. Two-way ANOVA was applied to analyze the data in panels (A–E), followed by Tukey’s multiple comparisons test for post hoc analysis. **P < 0.01. *ALKBH5*: AlkB homolog 5; ADR: Adriamycin; CCK-8: Cell counting kit-8; TUNEL: Transferase-mediated dUTP nick end labeling; NC: Negative control; qRT-PCR: Quantitative real-time polymerase chain reaction.

### RNA stability detection

To determine RNA stability, treated cells were seeded into 24-well plates for 24 h, then exposed to actinomycin D (5 µg/mL) for 0, 3, 6, 9, and 12 h. Cells were collected, and TUG1 levels were measured by qRT-PCR.

### Subcellular localization

Following the PARIS Kit instructions (Life Technologies, Carlsbad, CA, USA), ECA109 cell nuclei and cytoplasm were separated, and *TUG1* levels were quantified by qRT-PCR using U6 as a nuclear reference and GAPDH as a cytoplasmic reference. Primer sequences are listed in Table 2.

### Chromatin immunoprecipitation (ChIP) assay

ChIP was conducted on HL60 and HL60/ADR cells using the EZ-ChIP kit (Millipore). Chromatin cross-linked with formaldehyde was sonicated and immunoprecipitated with antibodies against EHMT2 (1:50, ab229455), H3K9me2 (1:30, ab32521), or IgG (1:50, ab172730) (all from Abcam). Purified chromatin was analyzed by qRT-PCR.

### Xenograft tumors in nude mice

Twenty-four male BALB/c-nude mice (6–8 weeks old, Shanghai SLAC Laboratory Animal Co., Ltd., Shanghai, China, SYXK [Shanghai] 2022-0012)were randomly divided into sh-NC and sh-ALKBH5 groups (*n* ═ 12). Experimenters were blinded to group assignments throughout. HL60/ADR cells stably transfected with sh-NC or sh-ALKBH5 were resuspended in PBS (2 × 10^ImEquation3^ cells/mL) and injected subcutaneously into the back of each mouse. Mice were euthanized if body weight loss exceeded 10% or if tumor diameter exceeded 1.5 cm. Seven days post-injection, ADR (3 mg/kg) was administered intraperitoneally once weekly for four weeks. Tumor growth was measured on days 7, 14, 21, and 28. Mice were euthanized by pentobarbital sodium injection (100 mg/kg), and tumors were collected for analysis.

### Immunohistochemistry (IHC) staining

Tumor tissues were fixed in 4% paraformaldehyde, embedded in paraffin, and sectioned at 5 µm. Sections were dewaxed, rehydrated, and blocked with 3% H_2_O_2_ for 20 min to inhibit endogenous peroxidase. They were then incubated with rabbit anti-Ki67 (1:1000, ab15580, Abcam) overnight at 4 ^∘^C, followed by goat anti-rabbit IgG (1:1000, ab6721, Abcam) for 30 min. Sections were dehydrated, sealed with neutral resin, and observed under a microscope.

### Bioinformatics

The subcellular localization of *TUG1* was predicted using the lncATLAS database (https://lncatlas.crg.eu/) [18], and its interaction with EHMT2 was predicted via the RNA–protein interaction database (http://rna.sysu.edu.cn/chipbase/) [19].

### Ethical statement

This study was approved by the ethics committee of the Sixth Affiliated Hospital of Wenzhou Medical University (Approval number: 2023LLW-053) and by the Institutional Animal Care and Use Committee of the same institution. All procedures conformed to the NIH Guidelines for the Care and Use of Laboratory Animals. Efforts were made to reduce animal numbers and suffering.

**Figure 3.**
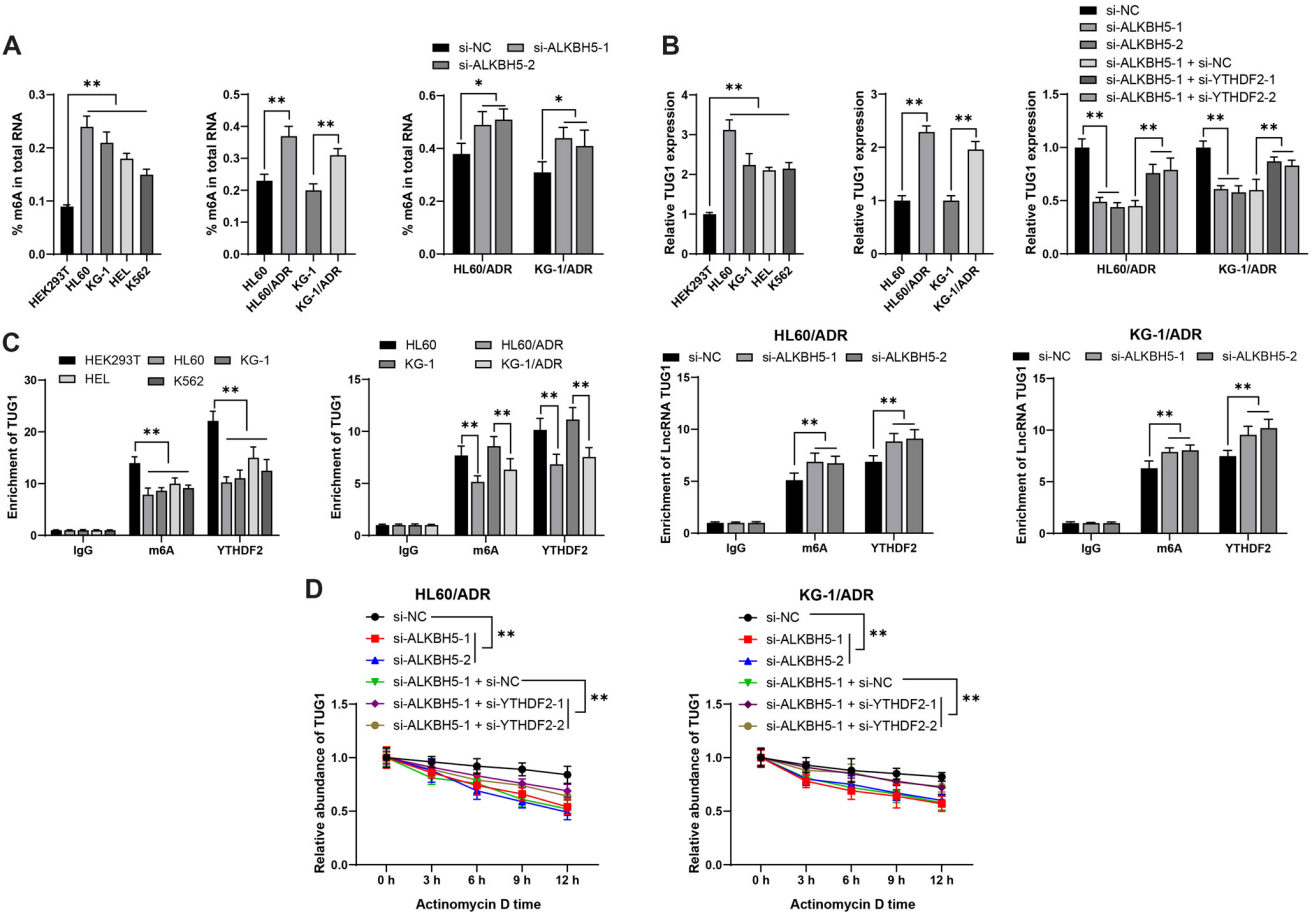
***ALKBH5* regulates *TUG1* expression in an m6A/YTHDF2-dependent manner.** Panel (A): The total m6A level in cells was measured. Panel (B): *TUG1* expression was assessed by qRT-PCR. Panel (C): The m6A modification level of *TUG1* and its binding with YTHDF2 were evaluated using RIP assays. Panel (D): *TUG1* stability was assessed following treatment with actinomycin D. All experiments were conducted independently in triplicate, with data presented as mean ± standard deviation. Two-way ANOVA was used for analysis in panels (C and D), with Tukey’s multiple comparisons test applied for post hoc analysis. *P < 0.05, **P < 0.01. *YTHDF2*: YTH N6-methyladenosine RNA binding protein F2; *TUG1*: Taurine upregulated 1; RIP: RNA immunoprecipitation; qRT-PCR: Quantitative real-time polymerase chain reaction; *ALKBH5*: AlkB homolog 5.

### Statistical analysis

Data were analyzed using SPSS 21.0 (IBM Corp., Armonk, NY, USA) and graphs generated with GraphPad Prism 8.0 (GraphPad Software Inc., San Diego, CA, USA). Results are presented as mean ± standard deviation. Normality and homogeneity of variance were checked. A *t*-test was used for two-group comparisons, while one-way or two-way ANOVA was used for multiple comparisons, followed by Tukey’s post hoc test. *P* < 0.05 was considered statistically significant, and *P* < 0.01 was considered highly significant.

## Results

### ALKBH5 is overexpressed in AML ADR-resistant cells

Compared with HEK293T cells, AML cells showed increased *ALKBH5* expression (*P* < 0.01, Figure 1A and 1B). Furthermore, AML ADR-resistant cell lines (HL60/ADR and KG-1/ADR) were established, showing significant *ALKBH5* overexpression (*P* < 0.05, Figure 1C and 1D). Additionally, the IC50 values of HL60/ADR and KG-1/ADR cells for ADR were higher than those of parental cells (*P* < 0.01, Figure 1E). Results from the CCK-8 assay indicated that 5 and 3 µM ADR were optimal for subsequent HL60 and KG-1 cell treatments. The colony formation assay suggested that ADR treatment enhanced resistant cell proliferation (*P* < 0.01, Figure 1F) and downregulated apoptosis compared to parental cells (*P* < 0.01, Figure 1G).

**Figure 4.**
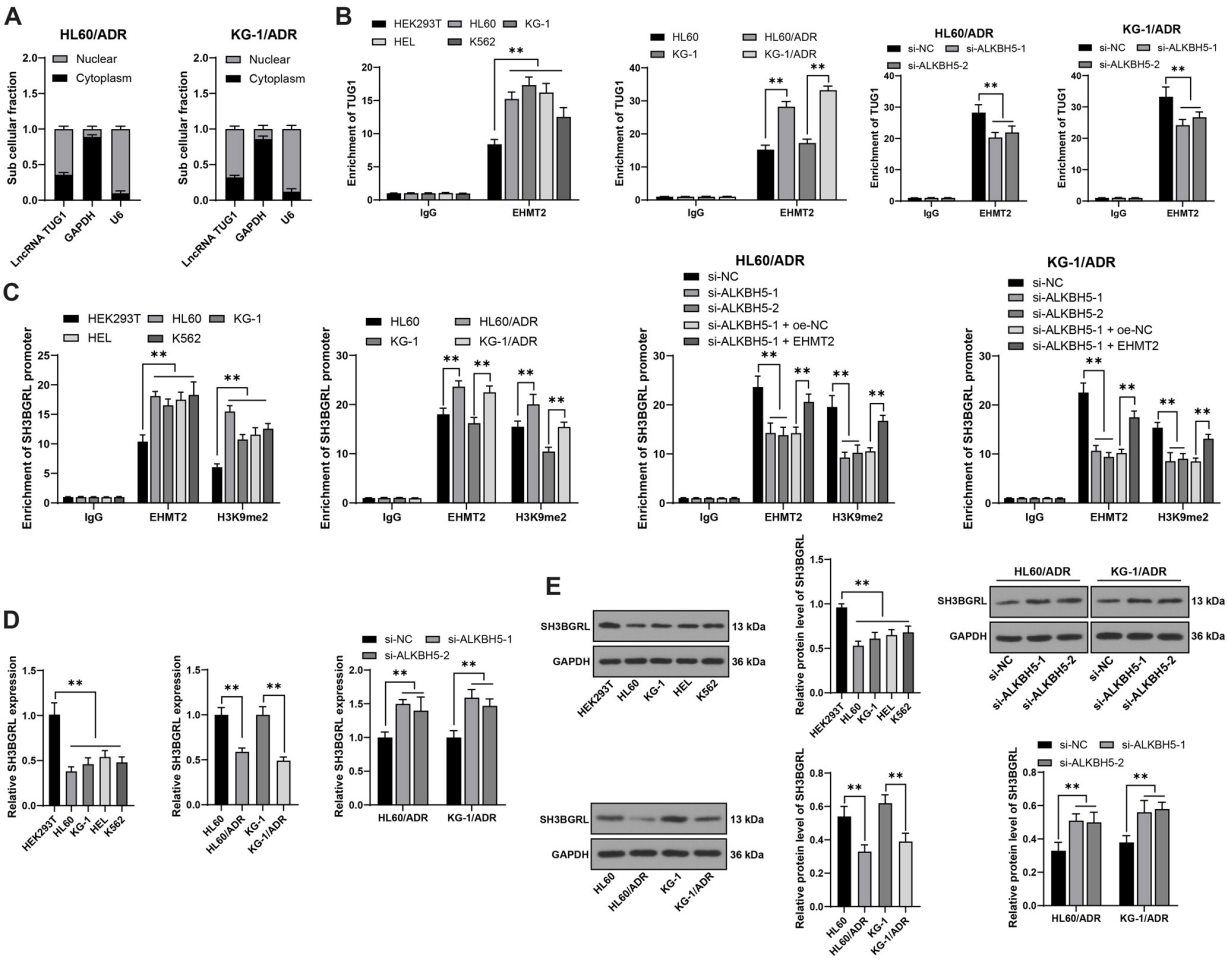
***TUG1* promotes H3K9me2 enrichment on the *SH3BGRL* promoter, thereby suppressing *SH3BGRL* expression.** Panel (A): *TUG1* localization was confirmed primarily in the cell nucleus through nuclear/cytoplasmic fractionation experiments. Panel (B): The interaction between *TUG1* and EHMT2 was validated via RIP assay. Panel (C): Enrichment of EHMT2 and H3K9me2 on the *SH3BGRL* promoter was assessed using ChIP assays. Panels (D and E): *SH3BGRL* expression was measured by qRT-PCR (D) and western blot analysis (E). All experiments were independently repeated three times, with data presented as mean ± standard deviation. Two-way ANOVA was used to analyze data in panels (A–C), while one-way or two-way ANOVA was applied for panels (D and E). Tukey’s multiple comparisons test was used for post hoc analysis. *P < 0.05, **P < 0.01. TUG1: Taurine upregulated 1; RIP: RNA immunoprecipitation; qRT-PCR: Quantitative real-time polymerase chain reaction; *SH3BGRL*: SH3 domain-binding glutamate-rich protein-like; *EHMT2*: Euchromatic histone lysine methyltransferase 2; ChIP: Chromatin immunoprecipitation.

**Figure 5.**
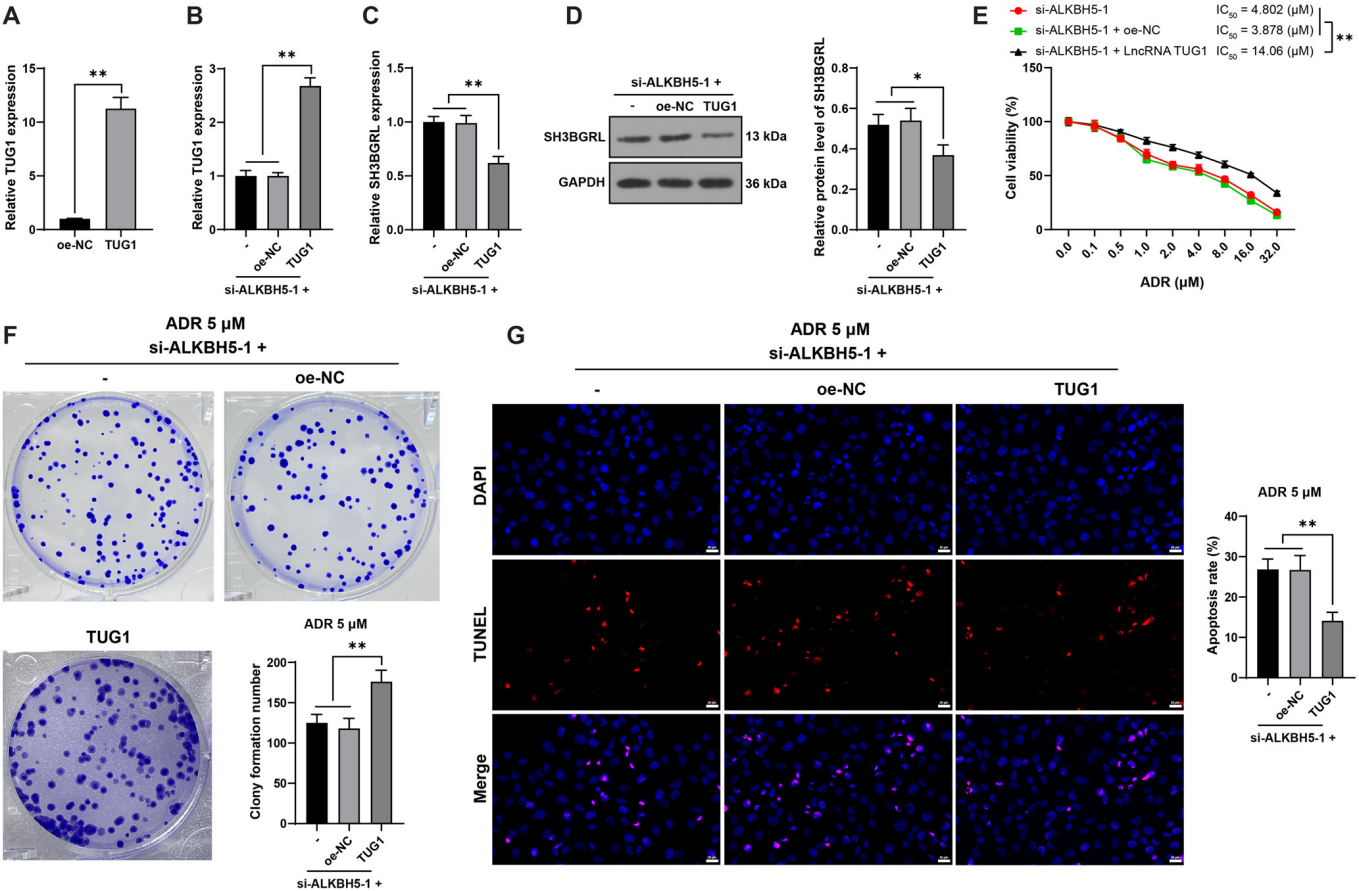
***TUG1* overexpression partially reverses the inhibitory effect of *ALKBH5* knockdown on ADR resistance**. Combined experiments were conducted in which oe-TUG1 and si-ALKBH5-1 were transfected into HL60/ADR cells, with oe-NC serving as the control. Panel (A): Transfection efficiency was assessed by qRT-PCR. Panel (B): *TUG1* expression was validated by qRT-PCR. Panels (C and D): *SH3BGRL* expression was measured using qRT-PCR (C) and western blot analysis (D). Panel (E): Cell viability and the IC50 for ADR were evaluated using the CCK-8 assay. Panel (F): Cell proliferation was assessed through a colony formation assay. Panel (G): Apoptosis was measured using TUNEL staining. All experiments were independently repeated three times, with data presented as mean ± standard deviation. One-way ANOVA was used to analyze data in panels (A–D, F, and G), while two-way ANOVA was applied for panel (E). Tukey’s multiple comparisons test was used for post hoc analysis. *P < 0.05, **P < 0.01. *TUG1*: Taurine upregulated 1; *ALKBH5*: AlkB homolog 5; *SH3BGRL*: SH3 domain-binding glutamate-rich protein-like; ADR: Adriamycin; CCK-8: Cell counting kit-8; TUNEL: Transferase-mediated dUTP nick end labeling; qRT-PCR: Quantitative real-time polymerase chain reaction; NC: Negative control.

**Figure 6.**
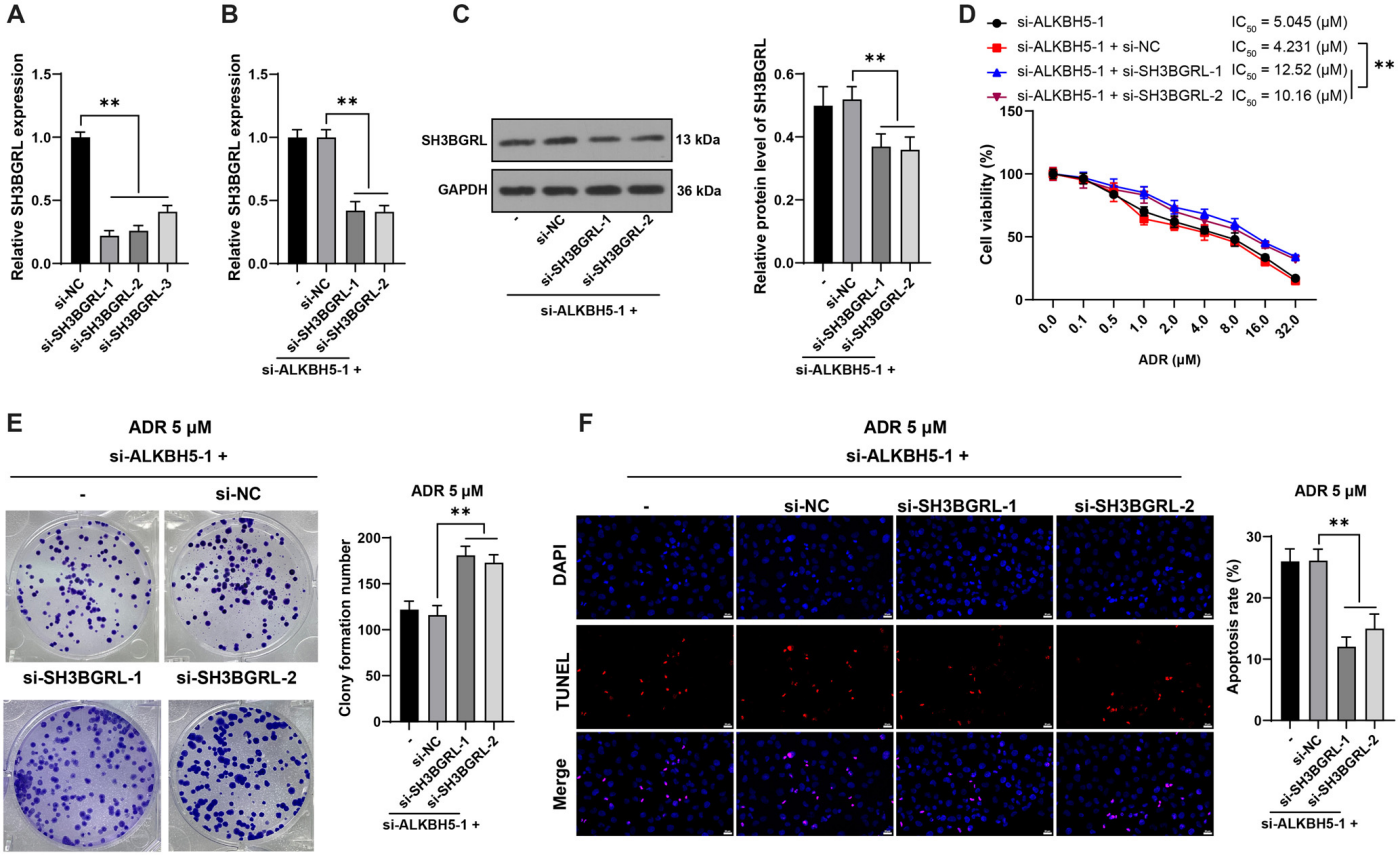
***SH3BGRL* knockdown partially reverses the inhibitory effect of *ALKBH5* knockdown on ADR resistance in HL60/ADR cells**. si-SH3BGRL constructs (si-SH3BGRL-1, si-SH3BGRL-2, and si-SH3BGRL-3) were transfected into HL60/ADR cells, with si-NC as the control. Panel (A): Transfection efficiency was evaluated by qRT-PCR, with si-SH3BGRL-1 and si-SH3BGRL-2 selected for subsequent experiments due to their higher transfection efficiency. Panels (B and C): *SH3BGRL* expression was measured by qRT-PCR (B) and western blot analysis (C). Panel (D): Cell viability and the IC50 for ADR were assessed using the CCK-8 assay. Panel (E): Cell proliferation was examined through colony formation assays. Panel (F): Apoptosis was measured using TUNEL staining. All experiments were independently repeated three times, with data presented as mean ± standard deviation. One-way ANOVA was used for data analysis in panels (A–C, E, and F), while two-way ANOVA was applied for panel (D). Tukey’s multiple comparisons test was used for post hoc analysis. *P < 0.05, **P < 0.01. *ALKBH5*: AlkB homolog 5; *SH3BGRL*: SH3 domain-binding glutamate-rich protein-like; ADR: Adriamycin; CCK-8: Cell counting kit-8; TUNEL: Transferase-mediated dUTP nick end labeling; qRT-PCR: Quantitative real-time polymerase chain reaction; NC: Negative control.

### Inhibition of *ALKBH5* downregulates ADR resistance of HL60/ADR and KG-1/ADR cells

To validate *ALKBH5*’s role in AML resistance to ADR, si-ALKBH5-1, si-ALKBH5-2, and si-ALKBH5-3 were transfected into HL60/ADR and KG-1/ADR cells to suppress *ALKBH5* expression (*P* < 0.05, Figure 2A). Si-ALKBH5-1 and si-ALKBH5-2 were selected for further experiments due to their higher transfection efficiency. Silencing *ALKBH5* (*P* < 0.01, Figure 2A and 2B) decreased the IC50 values of HL60/ADR and KG-1/ADR cells for ADR (*P* < 0.01, Figure 2C), reduced cell proliferation (*P* < 0.05, Figure 2D), and increased apoptosis (*P* < 0.01, Figure 2E). These findings indicate that *ALKBH5* silencing can downregulate ADR resistance in HL60/ADR and KG-1/ADR cells.

### *ALKBH5* reduces m6A modification to stabilize *TUG1* expression in a YTHDF2-dependent manner

**Figure S1.**
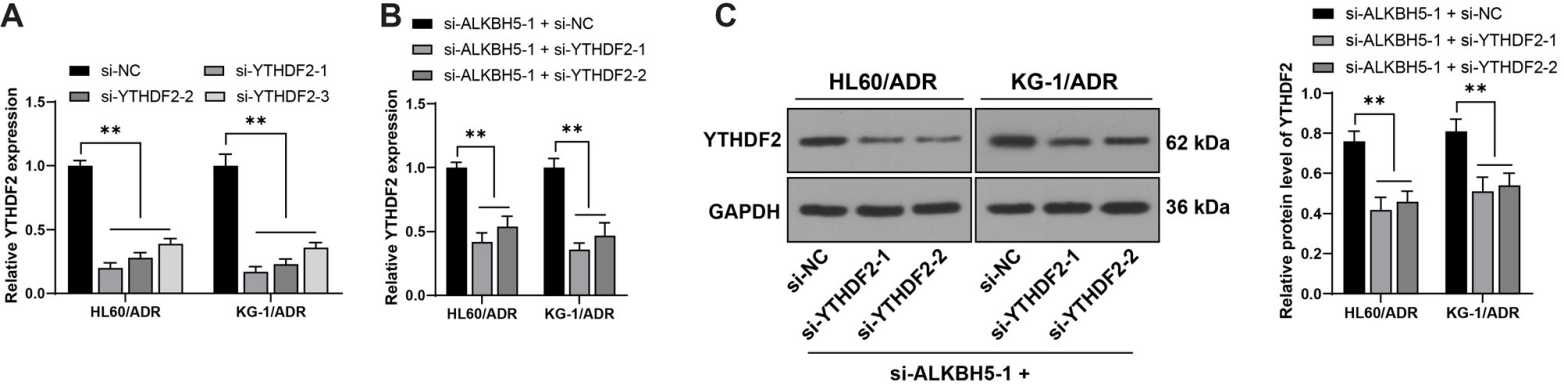
**si-YTHDF2 constructs (si-YTHDF2-1, si-YTHDF2-2, and si-YTHDF2-3) were transfected into HL60/ADR and KG-1/ADR cells, with si-NC as the control, for combination experiments with si-ALKBH5-1**. Panel (A): Transfection efficiency was measured by qRT-PCR, with the most effective siRNAs (si-YTHDF2-1 and si-YTHDF2-2) selected for further experiments. Panels (B and C): *YTHDF2* expression was assessed by qRT-PCR (B) and western blot analysis (C). All data are presented as mean ± standard deviation. One-way or two-way ANOVA was used to analyze the data in panels (A and B), while two-way ANOVA was applied to panel (C). Tukey’s multiple comparisons test was used for post hoc analysis. **P < 0.01. *YTHDF2*: YTH N6-methyladenosine RNA binding protein F2; *ALKBH5*: AlkB homolog 5; NC: Negative control; ADR: Adriamycin; qRT-PCR: Quantitative real-time polymerase chain reaction.

*ALKBH5*, a critical m6A demethylase, modulates downstream gene expression by reducing m6A modification [20]. The total m6A level in AML cells was elevated and further overexpressed in drug-resistant cells (*P* < 0.05, Figure 3A). Silencing *ALKBH5* increased m6A levels in HL60/ADR and KG-1/ADR cells (*P* < 0.05, Figure 3A). *TUG1* was overexpressed in AML [21], and YTHDF2, which binds to m6A-modified mRNA, inhibited downstream gene expression [7]. We hypothesized that ALKBH5 may promote *TUG1* expression by reducing m6A modification in a YTHDF2-dependent manner. *TUG1* expression was upregulated in AML cell lines, especially in resistant cells (*P* < 0.01, Figure 3B). Silencing *ALKBH5* downregulated *TUG1* expression (*P* < 0.01, Figure 3B). Moreover, m6A modification and YTHDF2 enrichment on *TUG1* were reduced in AML cells, and further diminished in drug-resistant cells (*P* < 0.01, Figure 3C), while silencing *ALKBH5* enhanced both (*P* < 0.01, Figure 3C). Following actinomycin D treatment, *ALKBH5* inhibition reduced TUG1 stability (*P* < 0.01, Figure 3D). Silencing both *YTHDF2* and *ALKBH5* in drug-resistant cells (*P* < 0.01, Figure S1A–S1C) improved *TUG1* stability (*P* < 0.01, Figure 3D) and expression (*P* < 0.01, Figure 3B), suggesting that *ALKBH5* stabilizes *TUG1* expression by reducing m6A modification in a YTHDF2-dependent manner.

### *TUG1* binds to EHMT2 to enhance H3K9me2 level on the *SH3BGRL* promoter, thereby inhibiting *SH3BGRL* expression

**Figure S2.**
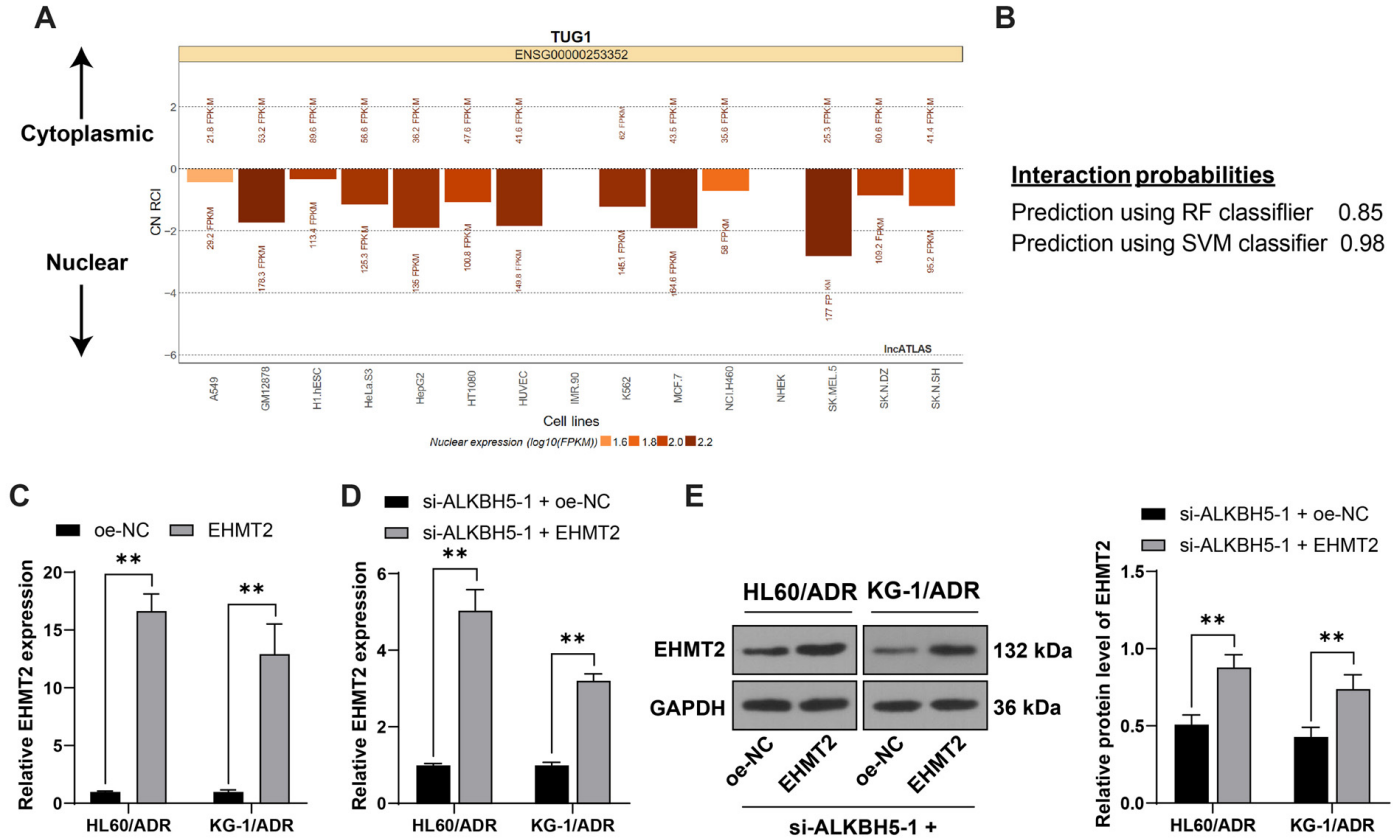
Panel (A): *TUG1*’s predominant nuclear localization was predicted using the lncATLAS database (https://lncatlas.crg.eu/). Panel (B): The interaction between *TUG1* and EHMT2 was confirmed through the RNA–protein interaction database (http://rna.sysu.edu.cn/chipbase/). Panels (C–E): oe-EHMT2 was transfected into HL60/ADR and KG-1/ADR cells, with oe-NC as the control. Transfection efficiency was evaluated by qRT-PCR (C), and *EHMT2* expression in cells was measured by qRT-PCR (D) and western blot analysis (E). All experiments were independently repeated three times, with data presented as mean ± standard deviation. Two-way ANOVA was used to analyze data in panels (C–E), followed by Tukey’s multiple comparisons test for post hoc analysis. **P < 0.01. *TUG1*: Taurine upregulated 1; *EHMT2*: Euchromatic histone lysine methyltransferase 2; ADR: Adriamycin; qRT-PCR: Quantitative real-time polymerase chain reaction; NC: Negative control.

The lncATLAS database (https://lncatlas.crg.eu/) [18] predicted *TUG1* localization in the nucleus (Figure S2A), confirmed by nuclear/cytoplasmic fractionation experiments (Figure 4A), suggesting that *TUG1* acts as a transcriptional regulator in the nucleus. EHMT2, a key histone demethylase, can suppress downstream gene expression via H3K9me2 [22]. *TUG1* was shown to bind to EHMT2 through the RNA–protein interaction database (Figure S2B). RIP assays indicated that *TUG1* binds EHMT2 in AML cells, with increased binding in HL60/ADR and KG-1/ADR cells, which was reduced upon *ALKBH5* silencing (*P* < 0.01, Figure 4B). Prior studies reported SH3BGRL downregulation in AML subjects [23]. *TUG1* potentially regulates SH3BGRL expression via EHMT2-mediated H3K9me2 modification. ChIP assays demonstrated EHMT2 and H3K9me2 enrichment on the SH3BGRL promoter, suppressed upon *ALKBH5* silencing (*P* < 0.01, Figure 4C). *EHMT2* overexpression (*P* < 0.01, Figure S2C and S2D) increased EHMT2 and H3K9me2 enrichment on the SH3BGRL promoter (*P* < 0.01, Figure 4C), and *SH3BGRL* expression was downregulated in AML cell lines, further diminished in resistant lines, but upregulated with *ALKBH5* silencing (*P* < 0.01, Figure 4D and 4E). Overall, *TUG1* binds EHMT2 to enhance H3K9me2 modification on the *SH3BGRL* promoter, inhibiting SH3BGRL transcription and protein expression.

### *TUG1* overexpression partially reverses the suppressive effect of *ALKBH5* knockdown on HL60/ADR drug resistance

*TUG1* overexpression (*P* < 0.01, Figure 5A and 5B) and *ALKBH5* silencing in HL60/ADR cells were achieved through transfection. Compared with the si-ALKBH5-1 group, the si-ALKBH5-1 + LncRNA TUG1 group exhibited reduced *SH3BGRL* expression (*P* < 0.05, Figure 5C and 5D), increased IC50 for ADR (*P* < 0.01, Figure 5E), enhanced cell proliferation (*P* < 0.01, Figure 5F), and inhibited apoptosis (*P* < 0.01, Figure 5G). These findings indicate that *TUG1* overexpression reverses the suppressive effects of *ALKBH5* knockdown on HL60/ADR drug resistance.

### *SH3BGRL* knockdown partially reverses the suppressive effect of *ALKBH5* knockdown on HL60/ADR drug resistance

Following *SH3BGRL* downregulation (*P* < 0.01, Figure 6A–6C) and *ALKBH5* silencing in HL60/ADR cells, *SH3BGRL* knockdown increased the IC50 of HL60/ADR cells to ADR (*P* < 0.01, Figure 6D), promoted cell proliferation (*P* < 0.01, Figure 6E), and inhibited apoptosis (*P* < 0.01, Figure 6F), indicating that *SH3BGRL* knockdown reverses the suppressive effect of *ALKBH5* knockdown on HL60/ADR drug resistance.

### *ALKBH5* silencing suppresses AML cell drug resistance to ADR *in vivo*

**Figure 7.**
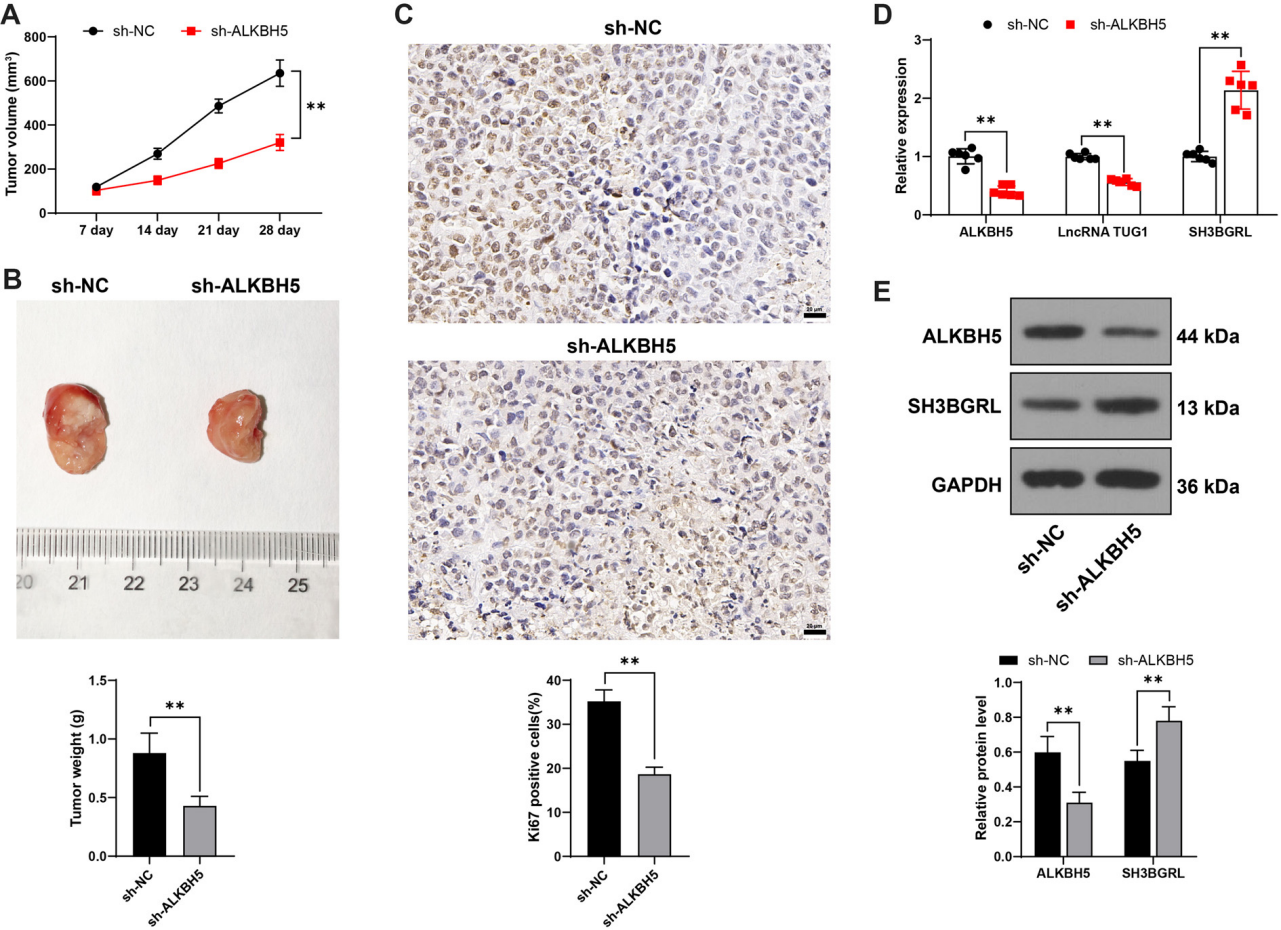
***ALKBH5* silencing reduces ADR resistance in AML cells *in vivo***. HL60/ADR cells infected with either sh-NC or sh-ALKBH5 were implanted into nude mice to establish xenograft tumor models, followed by ADR treatment. Panels (A and B): Tumor volume (A) and weight (B) were recorded. Panel (C): Ki67-positive cells were detected via IHC staining. Panel (D): Expressions of *ALKBH5*, *TUG1*, and *SH3BGRL* in tumor tissues were measured by qRT-PCR. Panel (E): Expressions of *ALKBH5* and SH3BGRL in tumor tissues were analyzed by western blot. *N* ═ 6 for each group, with data presented as mean ± standard deviation. A *t-*test was used for data analysis in panels (B and C), while two-way ANOVA was applied for panels (A, D, and E), followed by Tukey’s multiple comparisons test for post hoc analysis. *P < 0.05, **P < 0.01. *TUG1*: Taurine upregulated 1; *ALKBH5*: AlkB homolog 5; ADR: Adriamycin; *SH3BGRL*: SH3 domain-binding glutamate-rich protein-like; AML: Acute myeloid leukemia; qRT-PCR: Quantitative real-time polymerase chain reaction; IHC: Immunohistochemistry; NC: Negative control.

HL60/ADR cells with stable *ALKBH5* downregulation were injected into nude mice to establish xenograft tumor models, followed by ADR treatment. *ALKBH5* silencing inhibited tumor growth (*P* < 0.01, Figure 7A and 7B) and reduced the Ki67 positive rate in tumor tissues (*P* < 0.01, Figure 7C). qRT-PCR and western blot analysis showed that, upon *ALKBH5* silencing in tumor tissues, *ALKBH5* and *TUG1* were decreased, while SH3BGRL expression was upregulated (*P* < 0.01, Figure 7D and 7E), suggesting that ALKBH5 silencing suppresses AML cell drug resistance to ADR *in vivo.*

## Discussion

AML is a heterogeneous malignancy with increasing incidence and mortality rates. Treatment options for AML remain limited, and available regimens can often lead to unfavorable outcomes [24]. Currently, anthracycline-based drugs, including ADR, represent a mainstay treatment approach. However, drug resistance to ADR compromises treatment efficacy, increases relapse rates, and worsens prognosis [25]. Emerging evidence shows that RNA modifications play a role in cancer cell proliferation, metastasis, and immune response, potentially serving as therapeutic targets [26]. RNA demethylase *ALKBH5* has been identified as a viable target in cancer treatment due to its role in chemotherapy resistance through m6A demethylation [27]. In this study, we examined the relationship between *ALKBH5* and ADR resistance in AML, focusing on the *TUG1/EHMT2/SH3BGRL* pathway in a YTHDF2-dependent manner.

*ALKBH5*-mediated m6A modification plays a crucial role in various human cancers [28]. *ALKBH5* enhances cancer cell proliferation, reverses DNA damage, and inhibits apoptosis, leading to reduced ADR sensitivity, as observed in breast cancer [29]. Interestingly, *ALKBH5* overexpression can accelerate AML progression by activating oncogenes and promoting leukocyte differentiation, contributing to poor prognosis [30]. Our key finding was that *ALKBH5* was overexpressed in AML ADR-resistant cells, while *ALKBH5* inhibition reduced ADR resistance. Increased *ALKBH5* expression was linked to a poor prognosis and heightened cell resistance to ADR [31]. Taken together, our findings support that *ALKBH5* enhances AML cell resistance to ADR.

Mechanistically, m6A modification can serve as an upstream regulator of related lncRNAs in cancer [32]. The m6A reader YTHDF2 is known to inhibit apoptosis and promote drug resistance by regulating downstream target protein levels in a YTHDF2-dependent manner [33]. This study found that *ALKBH5* stabilizes *TUG1* expression by reducing m6A modification in a YTHDF2-dependent manner. These findings suggest that *ALKBH5* stabilizes *TUG1* expression through YTHDF2, thereby influencing ADR resistance in HL60/ADR and KG-1/ADR cells.

**Figure 8.**
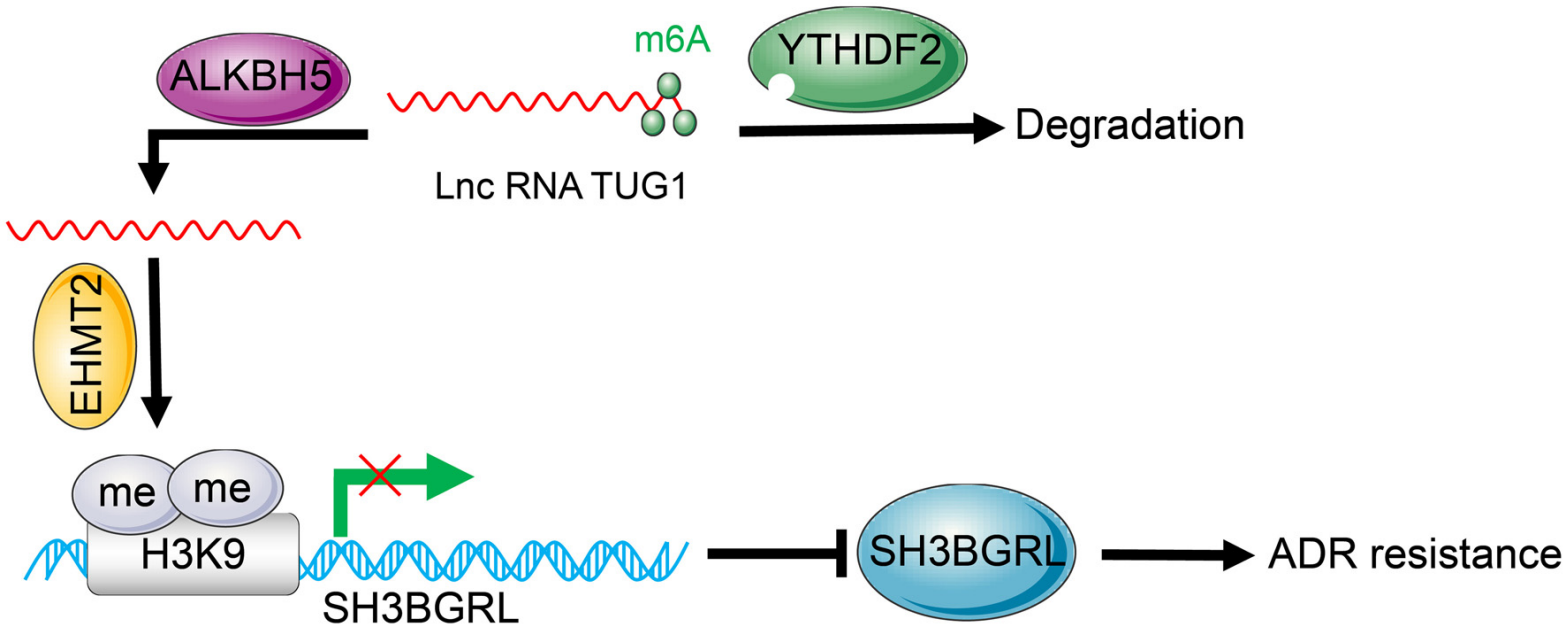
**ALKBH5 upregulates *TUG1* expression in a YTHDF2-dependent manner.** *TUG1*, in turn, suppresses *SH3BGRL* transcription by promoting H3K9me2 enrichment on the *SH3BGRL* promoter region, thereby enhancing ADR resistance in AML cells. *TUG1*: Taurine upregulated 1; *YTHDF2*: YTH N6-methyladenosine RNA binding protein F2; *ALKBH5*: AlkB homolog 5; ADR: Adriamycin; *SH3BGRL*: SH3 domain-binding glutamate-rich protein-like; AML: Acute myeloid leukemia.

Nuclear lncRNAs can influence disease progression through stable transcriptional regulatory mechanisms [34]. *TUG1* has been identified in the nuclei of multiple myeloma cells [35], which aligns with our findings. Evidence shows that *TUG1* upregulation enhances ADR resistance in several cancers [21, 36]. Motivated by this, we investigated *TUG1*’s potential role in AML. Our results showed that *TUG1* binds to EHMT2, increasing H3K9me2 levels on the *SH3BGRL* promoter, thereby inhibiting *SH3BGRL* transcription and protein expression. To validate our findings, we overexpressed *TUG1* in ADR-resistant AML cells and observed that *TUG1* overexpression reversed the effects of *ALKBH5* knockdown on ADR resistance. *TUG1* was abundantly expressed in AML and was associated with increased cell viability and reduced apoptosis [37]. Importantly, *TUG1* deficiency enhanced ADR sensitivity and promoted apoptosis in AML cells [38]. Furthermore, when *SH3BGRL* was downregulated, the effect of *ALKBH5* knockdown on ADR resistance was also reversed. *SH3BGRL*, which is underexpressed in acute promyelocytic leukemia, is known to enhance survival rates when activated by certain anti-tumor therapies [39]. When *SH3BGRL* expression increased in the tumor environment, ADR resistance diminished, and treatment efficacy improved [40]. Therefore, both *TUG1* overexpression and *SH3BGRL* knockdown contribute to enhanced ADR resistance in AML cells.

Clinically, drug resistance is a major factor in AML treatment failure [16]. Understanding the molecular mechanisms underlying drug resistance is thus crucial for improving AML treatment outcomes. Our data suggest that *ALKBH5* removes m6A modification to stabilize *TUG1* expression through YTHDF2, promoting the interaction between *TUG1* and EHMT2. This leads to increased H3K9me2 modification on the *SH3BGRL* promoter, reducing *SH3BGRL* expression and enhancing ADR resistance in AML cells (Figure 8). These findings provide promising insights for future AML treatment strategies and offer a reference for optimizing therapy in AML patients. However, our study has limitations. We found that *ALKBH5* expression was elevated in AML-resistant cells, along with an increase in total intracellular m6A levels, consistent with recent studies [41]. This contradictory finding underscores the complexity of m6A modifications in AML cell regulation and drug resistance. Our study focused solely on the *ALKBH5/TUG1/SH3BGRL* pathway, excluding other downstream targets of ALKBH5. Future studies should explore additional regulatory mechanisms of m6A modifications affecting ADR resistance in AML cells. Similarly, as EHMT2 is an important histone methyltransferase, it may regulate other downstream genes beyond H3K9me2 modification on the *SH3BGRL* promoter. These mechanisms warrant further investigation in future research.

## Conclusion

In conclusion, our findings indicate that *ALKBH5* is overexpressed in AML and contributes to ADR resistance by inhibiting m6A modification, thereby promoting *TUG1* expression in a YTHDF2-dependent manner. This, in turn, increases H3K9me2 enrichment on the *SH3BGRL* promoter, suppressing *SH3BGRL* expression and enhancing ADR resistance in AML cells. These results suggest a potential therapeutic strategy for AML management.

## Supplemental data

## Data Availability

The data that support the findings of this study are available from the corresponding author upon reasonable request.
